# Activity Against *Pythium insidiosum* and Isolated Constituents of *Cordia insignis* Roots

**DOI:** 10.1002/cbdv.202502503

**Published:** 2025-12-19

**Authors:** Rennan Carlos de Oliveira, Kheytiany Hellen da Silva Lopes, Mariele Rondon Santos Gonçalves, Mario Geraldo de Carvalho, Yasmin Sena de Amorin Dias da Silva, Leonardo Gomes de Vasconcelos, Paulo Teixeira de Sousa Júnior, Ivana Maria Póvoa Violante, Tereza Auxiliadora Nascimento Ribeiro

**Affiliations:** ^1^ Federal University of Mato Grosso Cuiabá Mato Grosso Brazil; ^2^ National Pantanal Research Institute Universidade Federal de Mato Grosso Cuiabá Mato Grosso Brazil; ^3^ Federal Rural University of Rio de Janeiro Seropédica Rio de Janeiro Brazil; ^4^ University of Cuiabá Cuiabá Mato Grosso Brazil

**Keywords:** oomycete, phitiosis, phytochemistry, roots, structure elucidation

## Abstract

Pythiosis, caused by the oomycete *Pythium insidiosum*, is a worrying disease with its challenging treatment due to the phylogeny of the pathogen. Natural products are a promising alternative in the treatment of diseases. *Cordia insignis* (Boraginaceae) is a poorly studied species, but *Cordia* species exhibit broad bioactivity. This study investigated phytochemically the roots of the species and evaluated the anti‐oomycete activity of the crude extract (CE) and fractions against *P. insidiosum*. Seven compounds were isolated and identified, including β‐sitosterol, stigmasterol, campesterol, β‐sitosterol‐3‐*O*‐β‐d‐glucopyranosyl, tridecanoic acid, scoparone, and gracicleistanthoside. The steroids, saponin and coumarin, were reported of leaves and branches of *C. insignis*; the fatty acid and glycoside are being first reported for the genus. The oomyceticidal assays revealed promising potential, with the hexane fraction (FH) being the most potent (minimum inhibitory concentration [MIC]/minimum oomicidal concentration [MOC] of 15.625 µg/mL). In conclusion, this study expands the chemotaxonomic knowledge of *C. insignis* and highlights the pharmacological potential of its roots against pythiosis.

## Graphical abstract

This study investigates the roots of *Cordia insignis* in search of novel treatments for pythiosis, caused by the oomycete *Pythium insidiosum*. Seven compounds are isolated, including steroids, a saponin, a coumarin, a fatty acid, and a glycoside, the latter two are reported for the first time in the genus. The crude extract and fractions exhibit promising anti‐oomycete activity, with the hexane fraction is the most potent (MIC/MOC of 15.625 µg/mL), highlighting the pharmacological potential of *C. insignis* against pythiosis.

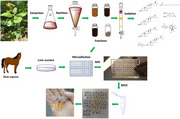

## 1 Introduction

Pythiosis is a severe disease caused by the oomycete *Pythium insidiosum*. The disease has a high overall mortality rate of 28% across all affected hosts and has been increasingly reported worldwide, particularly prevalent in tropical and subtropical regions [1]. It leads to significant health and economic consequences, notably in equine management, due to its challenging nature to treat and potential fatality if not promptly treated [2]. Despite having mycelium similar to fungi, *P. insidiosum's* phylogenetic characteristics distinguish it from true fungi, as it does not have chitin in its cell wall and some enzymes responsible for the synthesis of ergosterol [3]. This absence of ergosterol has proven to be a barrier to treatment with conventional antifungals that target it [2].

Its diagnosis is slow, making early detection difficult [1], and conventional therapies, which consist of extensive surgeries, immunotherapy [4], and the use of chemotherapy [5], have proven ineffective. This limitation has driven the search for alternative treatments, such as the use of azithromycin [6]; the combination of thymol and carvacrol with antifungals and antibiotics [7] and, most notably, plant extracts [2], which have demonstrated promising therapeutic potential.

Natural bioactive compounds perform specific biological functions, such as regulating endogenous defense mechanisms and interacting with organisms like cancerous cells and infectious pathogens [8]. The use of these bioresources, due to their intrinsic potential, evidenced by promising results in activities such as antifungal and anti‐oomycete action [9], is vital for developing biorational pesticides, offering sustainable alternatives to synthetic agrochemicals. Coupled with this, the structural modification capability of substances obtained from natural sources stands out. Interesting results regarding structure–activity relationships, which are crucial for guiding the modification and future enhancement of these molecules, have been observed [10].

In the Brazilian Cerrado alone, around 600 medicinal plant species have been recorded [11], including the species *Cordia insignis*, a shrub of the genus *Cordia* L. (Boraginaceae), which grows 3–4 m tall and is distributed across much of Brazil and extends to the central portion of Bolivia [12]. Commonly known as “calção‐de‐velho” [13], its leaves are traditionally used in teas and in alcoholic extracts to treat general pain and rheumatism [14], whereas the root is used for conditions such as hemorrhoids, hypertension, arthritis, bloating, jaundice, and obesity [15].

Research into other species within the genus *Cordia* is crucial, moving beyond merely replicating the success of *Cordia verbenacea*, which serves as a model for prospecting innovative molecules from Brazil's rich biodiversity [16]. This strategic approach is vital for accelerating the discovery of new drug prototypes and harnessing the national biotechnological potential. In this context, the genus is considered highly promising due to the pharmacological potential of its understudied species [17]. For instance, *C. insignis* has already demonstrated antifungal activity against fungi such as *Candida albicans* and *Cryptococcus neoformans*. Previous phytochemical studies have revealed the presence of diverse compounds, including triterpenes, steroids, saponins, coumarins, phenylpropanoid derivatives, and ureido [18].

This study is justified by the known bioactivities of other *Cordia* L. species and the limited information available on *C. insignis*, particularly its roots. Therefore, the aim was to investigate the phytochemistry of *C. insignis* roots, identify their chemical constituents, and assess the oomyceticidal activity of the crude extract (CE) and its fractions.

## 2 Results and Discussion

### 2.1 Isolated Constituents

**FIGURE 1:**
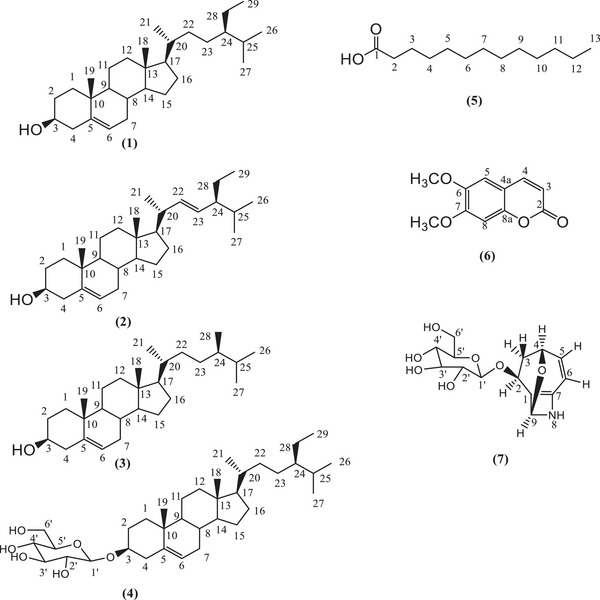
Structures of isolated compounds of *Cordia insignis*.

The phytochemical investigation of *C. insignis* roots resulted in the isolation and identification of 7 compounds (Figure 1), including three steroids, β‐sitosterol (**1**), stigmasterol (**2**), and campesterol (**3**); a saponin, β‐sitosterol‐3‐*O*‐β‐d‐glucopyranosyl (**4**) [18]; a fatty compound, tridecanoic acid [19, 20] (**5**); a coumarin, scoparone (**6**) [18], and an unusual glycoside, gracicleistanthoside (**7**) [21], whose structures were compared with data reported in the literature.

Compounds **1**, **2**, **3**, and **4** were identified as the major constituents of the hexane fraction (FH). Isolated as a white amorphous solid, Compounds **1**, **2**, and **3** are soluble in DCM, whereas Compound **4** is soluble in DCM/MeOH. Previous studies have reported that these phytosterols exhibit analgesic effects [22, 23], whereas anthelmintic and antimutagenic properties are specific to β‐sitosterol [22]. Saponin (Compound **4**) demonstrated moderate antibacterial activity against *Pseudomonas aeruginosa*, *Escherichia coli*, *Enterococcus faecalis*, and *Staphylococcus aureus* [24].

**FIGURE 2:**
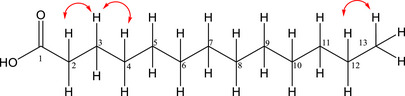
1H–1H COSY correlations of Compound **5**.

**FIGURE 3:**
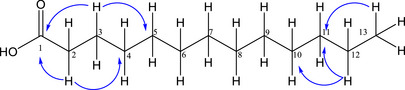
HMBC correlations of Compound **5**.

Compound **5**, isolated from the FH as a yellow viscous material, soluble in dichloromethane, was identified as an unusual fatty acid due to its odd number of carbon atoms. Although lipid characterization is most commonly performed by GC–MS, its main structural features were identified by NMR, revealing characteristic signals of methylene, methyl and carbonyl carbons [19], and their respective hydrogens [20]. The COSY spectrum of Compound **5** (Figure 2) revealed couplings between H‐2 and H‐3, H‐3 and H‐4, and H‐12 and H‐13. The absence of correlation in the HSQC spectrum for C‐1 is consistent with its ^13^C chemical shift, confirming the presence of a quaternary carbon characteristic of a carbonyl group. In the HMBC spectrum (Figure 3), correlations were observed from H‐2 and H‐3 to C‐1, H‐2 to C‐4 and H‐3 to C‐5, in addition to H‐12 for C‐10 and H‐13 for C‐11. The absence of correlations between H‐13 and C‐1/C‐2 suggests an acyclic molecular structure.

Compound **6** was isolated as a white solid, soluble in DCM. Its ^1^H NMR spectrum exhibited two doublets with a coupling constant of 9.45 Hz, characteristic of the olefinic hydrogens H‐3 and H‐4 of coumarin, in addition to the two methoxylic hydrogens, which is consistent with the literature data. In the DEPTQ spectrum, five quaternary carbon signals were observed, one corresponding to a carbonyl carbon and the other four to substituted aromatic carbons. This compound, previously reported in the stem of *C. insignis*, is known for a broad spectrum of biological activities, including hepatoprotective [25], antiallergic [26], antitumor [27], anti‐inflammatory [28], and acaricidal [29] effects.

**FIGURE 4:**
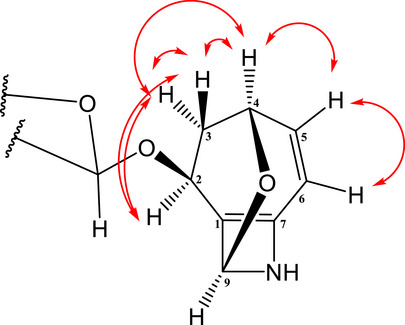
1H–1H COSY correlations of Compound **7**.

**FIGURE 5:**
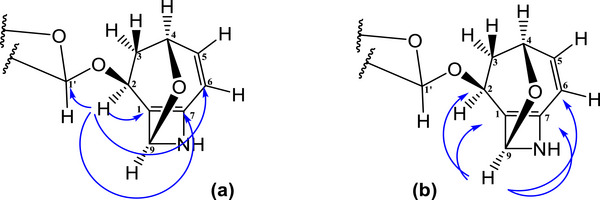
HMBC correlations of compound **7**: (a) correlations of H‐2; (b) correlations of H‐9.

Compound **7** was obtained as a yellowish viscous material soluble in methanol. Although its NMR spectra revealed the presence of multiple components, Compound **7** was identified as the major one. In the COSY spectrum of the aglycone (Figure 4), coupling between H‐2 and H‐3a/H‐3b, H‐3a/H‐3b and H‐4, H‐4 and H‐5, and H‐5 and H‐6 was observed. These couplings, together with the HMBC correlations of H‐2 to C‐1, C‐6 and C‐7 (Figure 5a), suggest a cyclic molecular structure. Additionally, the correlation of H‐9 to C‐1, C‐2, C‐6, and C‐7 (Figure 5b) suggests the presence of a second cycle in the molecule. These observations are consistent with literature data [20]. The correlation of H‐2 of the aglycone to C‐1′ (Figure 5a) of glucose confirms the identity of the compound as a glycoside.

This glycoside was originally reported in the roots of *Cleistanthus gracilis* [21] and was also identified in the species *Lithospermum erythrorhizon*, of the Boraginaceae family, where it exhibited anti‐inflammatory activity [30]. This is the first report of its isolation in the genus *Cordia* L. From a chemotaxonomic point of view, this study expands the phytochemical profile of *C. insignis*. The steroids, β‐sitosterol, stigmasterol and campesterol, the saponin β‐sitosterol‐3‐*O*‐β‐d‐glucopyranosyl, and the coumarin scoparone, are reported here for the first time in the roots of this species. In addition, tridecanoic acid and gracicleistanthoside represent the first reports for the genus.

### 2.2 Anti‐Oomycete Bioassay

The anti‐oomycete activity of the CE and its fractions was evaluated for the first time in this study against *P. insidiosum*, the etiological agent of pythiosis. The minimum inhibitory concentration (MIC) and minimum oomicidal concentration (MOC) values, obtained by the broth microdilution method, against two strains of the oomycete isolated from horses, are presented in Table 1.

**TABLE 1:** In vitro activity of *Cordia insignis* against two *Pythium insidiosum* isolates (4H and 364), tested in triplicate (*N* = 06).

		Number of readings (%) with the following MIC/CMR (µg/mL)										Minimum active range (µg/mL)	GC (µg/mL)
Extract/Fractions		1.95	3.91	7.81	15.62	31.25	62.5	125	250	500	1000		
**CE**	**MIC**	0	0	0	0	0	0	6 (100%)	0	0	0	125	125
	MOC	0	0	0	0	0	0	3 (50%)	0	3 (50%)	0	125–500	250
FH	**MIC**	0	0	0	6 (100%)	0	0	0	0	0	0	15.62	15.62
	**MOC**	0	0	0	6 (100%)	0	0	0	0	0	0	15.62	15.62
**FD**	**MIC**	0	0	0	0	0	3 (50%)	3 (50%)	0	0	0	62.5–125	88.39
	**MOC**	0	0	0	0	0	0	0	0	0	6 (100%)	1000	1000
**FA**	**MIC**	0	0	0	0	0	3 (50%)	3 (50%)	0	0	0	62.5–125	88.39
	**MOC**	0	0	0	0	0	3 (50%)	3 (50%)	0	0	0	62.5–125	88.39
**FHM**	**MIC**	0	0	0	0	3 (50%)	0	3 (50%)	0	0	0	31.25–125	62.5
	**MOC**	0	0	0	0	0	0	0	0	0	6 (100%)	1000	1000
**AMP B**	**MIC**	0	0	0	0	0	0	0	0	6 (100%)	0	500	—
	**MOC**	0	0	0	0	0	0	0	0	0	0	0	—

*Note: N*, total number of observations.

Abbreviations: AMP B, amphotericin B; CE, crude extract; FA, ethyl acetate fraction; FD, dichloromethane fraction; FH, hexanic fraction; FHM, hydromethanolic fraction; GM, geometric mean; MIC, minimum inhibitory concentration; MOC, minimum oomicidal concentration.

The fractions, CE, dichloromethane fraction (FD), FA, and hydromethanolic fraction (FHM) presented MIC, values of 125 µg/mL against strain 4H. For strain 364, the MICs were 125 µg/mL for CE, 62.5 µg/mL for FD and FA and 31.25 µg/mL for FHM. The FH fraction showed more promising activity, with an MIC of 15 625 µg/mL for both strains. MOC values followed a similar trend to the MIC. FH showed the strongest oomicidal effect, with an MOC of 15 625 µg/mL against both strains. For strain 4H, the MOCs were 125 µg/mL for CE and FA, and 1000 µg/mL for FD and FHM. For strain 364, the MOCs were 500 µg/mL for CE, 62.5 µg/mL for FA, and 1000 µg/mL for FD and FHM. When compared to values reported in the literature, the MIC and MOC results of FH, as well as the MOC of FA, against strain 364 are comparable to some commercial antifungal agents [31, 32, 33, 34].

Although the precise mechanism of action of natural products against *P. insidiosum* is not fully understood, the observed activity can be attributed to the presence of classes of metabolites such as phenolic compounds [35] found in more polar fractions, or terpenoids [36], present in the nonpolar fractions. The in vitro findings are promising for the development of new therapeutic alternatives, especially in relation to the FH. However, additional studies, including cytotoxicity assays, are needed to assess safety at the concentrations tested.

The CE was selected for screening due to two practical reasons: (i) exploratory screening of CEs is a well‐established approach in natural product research to identify potential bioactivities; and (ii) the yields of the isolated compounds were insufficient for the entire reproducible MIC/MOC testing. Additionally, synergistic effects among extract constituents are reported for various plant metabolites, and may contribute to the observed anti‐oomycete activity, supporting the rationale for evaluating the extract as a whole.

Although no chemical positive control was included in the experimental setup, our results were compared with MIC and inhibition values reported in the literature for *P. insidiosum* against agents such as mefenoxam, pyraclostrobin, and certain antibiotics. The activity levels of our extract fall within these published ranges, demonstrating that the observed effect is biologically relevant [37].

Even without performing mechanistic assays in this exploratory study, previous reports on natural metabolites with anti‐oomycete activity suggest multiple possible modes of action, including disruption of cell membrane integrity, induction of oxidative stress (ROS), and interference with cellular metabolism. These mechanisms are consistent with the chemical classes identified in our extract (sterols, saponins, fatty acids, coumarins), providing a plausible basis for the observed activity [38].

## 3 Conclusions

The phytochemical study performed allowed for the isolation and identification of seven compounds, which include the steroids β‐sitosterol, stigmasterol and campesterol, the saponin β‐sitosterol‐3‐*O*‐β‐d‐glucopyranosyl, and the coumarin scoparone. In addition to these, two substances that are being reported for the first time in the *Cordia* genus were identified as the fatty acid tridecanoic acid and the unusual glycoside gracicleistantoside. Although the steroids, saponin, and coumarin had already been previously identified in the species' leaves and branches, their presences were also confirmed in the roots. The anti‐omycete activity assay yielded promising results in the investigation of new therapies for the prevention and treatment of pythiosis. Both the CE and the fractions demonstrated an inhibitory action on the growth of the oomycete and an oomycidal action, with an emphasis on the FH fraction. Future studies will employ a combination of assays such as MTT, LDH release, and membrane integrity tests, as recommended for plant extracts, to avoid potential assay interference. Preliminary studies in this direction are underway. The identification of these metabolites, particularly those recently described for the species or genus, contributes to the chemotaxonomic understanding. Moreover, the observed anti‐omycete activity supports the pharmacological potential of *C. insignis* roots in the context of pythiosis treatment.

## 4 Experimental Section

### 4.1 General Experimental Procedures

Compounds’ identification was performed by one‐ and two‐dimensional nuclear magnetic resonance—1D and 2D NMR and/or comparison of retention factors with known standards and literature data. The experiments were acquired in CDCl_3_ and CD_3_OD in a Bruker Ascend 500 NMR spectrometer, operating at for ^1^H and for ^13^C at 500 and 125 MHz, respectively. Chemical shifts (*δ*) were expressed in ppm and coupling constants (*J*) in Hertz. The spectra were referenced with TMS. For column chromatography (CC), silica gel 60 with a particle size of 0.063–0.200 mm (70–230 mesh) (Merck) and Sephadex LH‐20 (Sigma‐Aldrich) were used, whereas for thin layer chromatography (TLC), silica gel 60 F254 (Merck) was employed. Compounds **1**, **2**, and **3** were also identified using a Shimadzu QP2030NX GC/MS equipped with a capillary column (30 m × 0.25 mm ID × 0.25 µm) containing a 5% diphenyl/95% dimethyl polysiloxane phase. Injector: 1 µL of sample solubilized in CHCl_3_ at a concentration of 1 mg/mL was injected, with helium as carrier gas, temperature 300°C, constant column flow rate of 1.0 mL/min, septum purge flow rate of 3 mL/min. Oven: Initial temperature 150°C, increment of 15°C/min up to 300°C and held for 10 min, increment of 10°C/min up to 325°C and held for 25.5 min, totaling 50 min of programming. Detector: Solvent Cut Time 4 min, starting scanning at 5 min and ending at 50 min, interface temperature of 325°C, ionization source temperature of 200°C, SCAN acquisition mode, scan speed 2000, scanning from *m/z* 40 to 600 Da.

### 4.2 Plant Materials

*C. insignis* (Sisgen code AF8CO57) was collected in Chapada dos Guimarães National Park in Mato Grosso State, Brazil in May 2023 (15°20′48.6″ S 55°51′20.3″ W). The species was identified by the botany technician Odil Pereira Dias. A voucher specimen (no. 46137) was stored at the Central Herbarium of the Universidade Federal do Mato Grosso, Cuiabá, Brazil.

### 4.3 Extraction and Isolation

The air‐dried and powdered roots (500 g) of *C. insignis* were extracted four times with a mixture of 80% ethanol in water (v/v) (1 L for each extraction) at room temperature for 7 days.

After removal of the solvent under reduced pressure, the crude hydroethanolic extract (CE, 50 g) was obtained. An aliquot of the CE (45 g) was suspended in 350 mL of a mixture of 80% methanol in water (v/v) and extracted with *n*‐hexane (FH, 1.25 g), dichloromethane (FD, 13.05 g), ethyl acetate (FA, 9.20 g), and the hydromethanolic residue (FHM, 21.50 g).

A portion of the FH (1.02 g) was subjected to CC with silica gel (70–230 mesh) using *n*‐Hex/DCM, DCM/EtOAc and EtOAc/MeOH (gradient) obtaining seven subfractions. The mixture of Compounds **1**, **2**, and **3** (43.5 mg) was obtained from Subfraction 4 and Compound **4** (32 mg) from Subfraction 7 after drying the solvent.

Subfraction 4 (256.2 mg) was then purified by CC on sephadex LH‐20 with DCM/MeOH (3:7 v/v) to obtain 7 subfractions. Subfraction 2 (129.3 mg) was chromatographed on CC with silica gel, eluted with DCM/EtOAc (9:1 v/v) and MeOH to provide 8 subfractions. Compound **5** (9.2 mg) was isolated from Subfraction 2 (17.4 mg) by preparative thin layer chromatography (PTLC) using DCM/EtOAc (95:5 v/v).

Fraction FD (10 g) was chromatographed on CC with silica gel, eluted with *n*‐hex/DCM, DCM/EtOAc, EtOAc/MeOH, and MeOH/H2O (gradient), resulting in eight subfractions. Subfraction 2 (294.4 mg) was subjected to CC on silica gel with *n*‐hex/DCM, DCM/EtOAc, EtOAc/MeOH (gradient), providing seven subfractions. Subfraction 4 (60.1 mg) was subjected to CC on silica gel with DCM/MeOH (gradient), resulting in six subfractions. Subfraction 3 (40 mg) was subjected to CC on sephadex LH‐20 (MeOH), yielding six subfractions. From Subfraction 2 (21.2 mg), 7 subfractions were obtained after elution on silica gel with *n*‐hex/DCM and DCM/MeOH (gradient). Compound **6** (4.4 mg) was isolated by PTLC from Subfraction 3 in DCM/MeOH (97:3 v/v).

Subfraction 6 (1.09 g) of FD was subjected to CC on sephadex LH‐20 with DCM/MeOH (3:7 v/v), yielding 8 subfractions. Of these, Subfraction 4 (629.1 mg) was subjected to silica gel in DCM/MeOH (gradient), yielding 8 subfractions. The sixth subfraction (326.7 mg) was subjected to sephadex LH‐20 (MeOH), yielding three subfractions. Subfraction 2 (133.5 mg) was divided into two parts and subjected to PTLC with DCM/MeOH (8:2 v/v). The second subfraction of part 2 (43.7 mg) was subjected to sephadex LH‐20 (MeOH), obtaining three subfractions. Subfraction 2 (29.8 mg) was pooled with the second subfraction of part 1 (20.6 mg) totaling 50.4 mg, and this was purified on CC with silica gel with EtOAc/MeOH (gradient) to obtain Compound **7** (17.9 mg).

**β‐sitosterol** (**1**): GC–MS (EI, 70 eV) *m/z* (%) 414 (M^+^ 100), 329 (80), 107 (70), 42 (66).

**Stigmasterol** (**2**): GC–MS (EI, 70 eV) *m/z* (%) 412 (74), 255 (60), 83 (98), 55 (100).

**Campesterol** (**3**): GC–MS (EI, 70 eV) *m/z* (%) 400 (M^+^ 100), 315 (82), 105 (80), 43 (74).

**β‐sitosterol‐3‐*O*‐β‐d‐glucopyranosyl** (**4**): ^1^H NMR (500 MHz, DMSO‐d_6_): *δ* 3.47 (1H, m, H‐3), 5.32 (1H, m, H‐6), 0.64 (3H, s, H‐18), 0.95 (3H, s, H‐19), 4.20 (1H, d, *J* = 7.85, H‐1′) 2.89 (1H, m, H‐2′), 3.11 (1H, m, H‐3′), 3.00 (1H, m, H‐4′), 3.05 (1H, m, H‐5′), 3.64 (1H, m, H‐6′a), 3.40 (1H, m, H‐6′b). ^13^H NMR (125 MHz, DMSO‐d_6_): *δ* 37.2 (C‐1), 29.7 (C‐2), 77.2 (C‐3), 38.7 (C‐4), 140.9 (C‐5), 121.7 (C‐6), 31.84 (C‐7), 31.88 (C‐8), 50.0 (C‐9), 36.6 (C‐10), 21.0 (C‐11), 39.6 (C‐12), 42.3 (C‐13), 56.6 (C‐14), 24.3 (C‐15), 28.2 (C‐16), 55.8 (C‐17), 12.1 (C‐18), 19.4 (C‐19), 35.9 (C‐20), 19.0 (C‐21), 33.7 (C‐22), 25.8 (C‐23), 45.5 (C‐24), 29.1 (C‐25), 19.5 (C‐26), 20.2 (C‐27), 23.0 (C‐28), 12.2 (C‐29), 101.2 (C‐1′), 73.9 (C‐2′), 77.3 (C‐3′), 70.5 (C‐4′), 77.2 (C‐5′), 61.4 (C‐6′).

**Tridecanoic acid** (**5**): ^1^H‐NMR (500 MHz, CDCl_3_) *δ*: 2.16 (2H, t, H‐2), 1.50 (2H, quint, H‐3), 1,18 (2H, H‐4), 1.14 (14H, brs, H‐5/11), 1.17 (2H, H‐12), 0,77 (3H, t, H‐13); DEPTQ (125 MHz, CDCl_3_) *δ*: 175.84 (C‐1), 33.92 (C‐2), 24.70 (C‐3), 28.92 (C‐4), 29.38 (C‐5), 29.37 (C‐6), 29.33 (C‐7), 29.20 (C‐8), 29.06 (C‐9), 29.05 (C‐10), 31.63 (C‐11), 22.40 (C‐12), 13.87 (C‐13).

**Scoparone** (**6**): ^1^H‐NMR (500 MHz, CDCl_3_) *δ*: 6.28 (1H, d, *J* = 9.45, H‐3), 7.62 (1H, *d*, *J* = 9.45, H‐4), 6.83 (1H, s, H‐5), 6.84 (1H, s, H‐8), 3.94 (3H, s, ─OCH_3_), 3.91 (3H, s, ─OCH_3_); DEPTQ (125 MHz, CDCl_3_) *δ*: 161.43 (C‐2), 113,57 (C‐3), 143.30 (C‐4), 111,45 (C‐4a), 107.99 (C‐5), 146.37 (C‐6), 152.87 (C‐7), 100.04 (C‐8), 150.05 (C‐8a), 56.39 (─OCH_3_), 56.37 (─OCH_3_).

**Gracicleistanthoside** (**7**): ^1^H‐NMR (500 MHz, CD_3_OD) *δ*: 4.93 (1H, ddd, H‐2), 2.27 (1H, m, H‐3a), 2.02 (1H, m, H‐3b), 4.36 (1H, q, H‐4), 6.19 (1H, dd, H‐5), 6.28 (1H, dd, H‐6), 5.50 (1H, brs, H‐9), 4.55 (1H, d, H‐1′), 3.35 (1H, m, H‐2′), 3.35 (1H, m, H‐3′), 3.27 (1H, d, H‐4′), 3.89 (1H, dd, H‐6′a), 3.66 (1H, dd, H‐6′b); ^13^C‐NMR (125 MHz, CD_3_OD) *δ*: 118.04 (C‐1), 72.51 (C‐2), 36.13 (C‐3), 65.39 (C‐4), 140.63 (C‐5), 127.74 (C‐6), 157.74 (C‐7), 96.80 (C‐9), 101.53 (C‐1′), 74.49 (C‐2′), 78.01 (C‐3′), 71.72 (C‐4′), 78.16 (C‐5′), 63.14 (C‐6′) (Supporting information).

### 4.4 Anti‐Oomycete Assay

For the bioassays of anti‐oomycete activity, two clinical isolates of *P. insidiosum* from equine were used: 4H (CBS 575.75; GenBank AB971178) and 364 (GenBank MT192482), provided by Laboratório de Pesquisa Micológica—LAPEMI of the Federal University of Santa Maria.

The inocula consisted of zoospores obtained by zoosporogenesis, according to the methodology adapted from Pereira et al. [39], at a concentration of 2.5–3 × 10^3^ zoospores/mL. These were diluted to 1:10 in RPMI 1640 broth with l‐glutamine and glucose and buffered to pH 7.0 with 0.165 M MOPS (assay medium). Susceptibility tests were performed by the broth microdilution method according to the Clinical and Laboratory Standards Institutes (CLSI) protocol M38‐A2, adapted by Pereira et al. [39]. The CE and fractions were diluted to final concentrations of 1000, 500, 250, 125, 62.5, 31.25, 15.625, 7.8125, 3.9062, and 1.9531 µg/mL. The MIC was determined after 48 h at 37°C, by observing the presence or absence of growth of the microorganism. The minimum oomycidal concentration (MOC) was determined by removing 0.1 mL aliquots from the wells where no growth was observed and transferring them to microtubes containing 0.9 mL of Sabouraud dextrose broth and incubating at 37°C for 48 h. The procedures were performed in triplicate.

### 4.5 Statistical Analysis

No data preprocessing (such as transformation or normalization) was performed in this study, owing to the observational and discrete nature of the data acquired via serial dilutions. Results were reported as the observed active concentration range and the geometric mean (GM). The percentage response frequency was calculated relative to the total number of observations (*N* = 6). Two microorganism strains were used, constituting the biological sample size (*n* = 2) for population inferences. Each assay was performed in triplicate to assess reproducibility, yielding a total of six raw observations (*N* = 6). Given the small biological sample size (*n* = 2), inferential statistical significance tests (such as ANOVA or *t*‐test) were not applied. Conclusions were therefore based solely on the comparison of descriptive statistics (mean and response frequency). Data tabulation and GM calculation were performed using Microsoft Excel software.

## Author Contributions

**Rennan Carlos de Oliveira**: writing, editing, and reviewing the manuscript. **Kheytiany Hellen da Silva Lopes**: writing and reviewing the manuscript. **Mariele Rondon Santos Gonçalves**: operation of spectroscopic experiment. **Mario Geraldo de Carvalho**: operation of spectroscopic experiment. **Yasmin Sena de Amorin Dias da Silva**: conducting the biological experiment. **Leonardo Gomes de Vasconcelos**: operation of spectroscopic and spectrometric experiment. **Paulo Teixeira de Sousa Júnior**: fundraising and review. **Ivana Maria Póvoa Violante**: supervision of the biological experiment. **Tereza Auxiliadora Nascimento Ribeiro**: project conception, guidance, fundraising, and review.

## Conflicts of Interest

The authors declare no conflicts of interest.

## Supporting information




**Supporting file 1**: cbdv70790‐sup‐0001‐SuppMat.pdf

## Data Availability

The data that support the findings of this study are available from the corresponding author upon reasonable request.
