# Supplementary material for: Activity Against Pythium insidiosum and Isolated Constituents of Cordia insignis Roots
Source: Chem Biodivers. 2025 Dec 19;23(2):e02503. doi: 10.1002/cbdv.202502503 (PMC12860512; doi:10.1002/cbdv.202502503)

Figure 1. Total ion chromatogram (GC/MS) of compounds 1, 2 and 3.

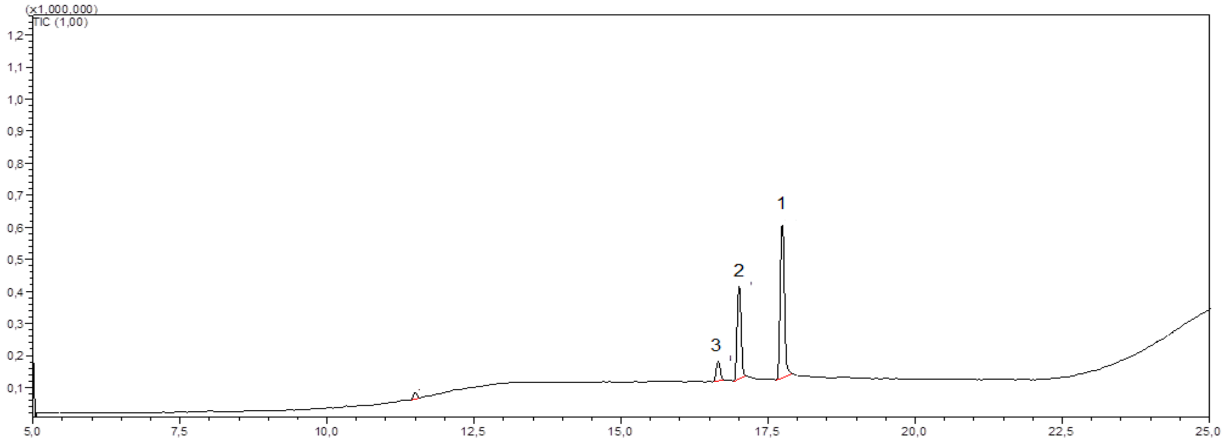

Figure 2. Mass spectrum of compound 1.

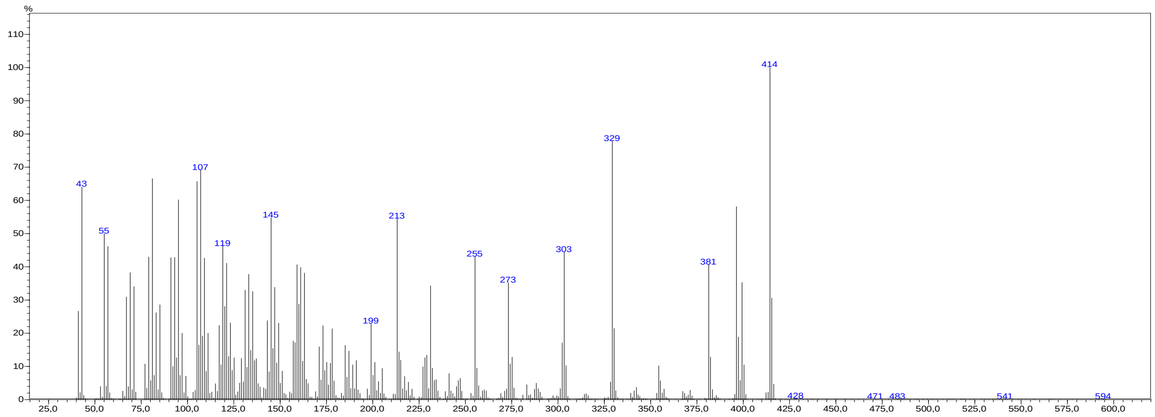

Figure 3. Mass spectrum of compound 2.

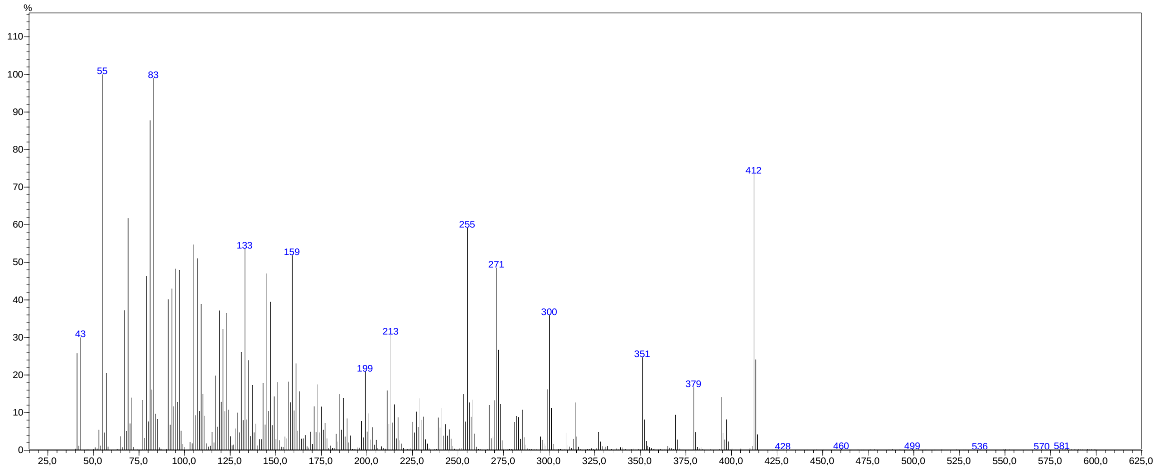

Figure 4. Mass spectrum of compound 3.

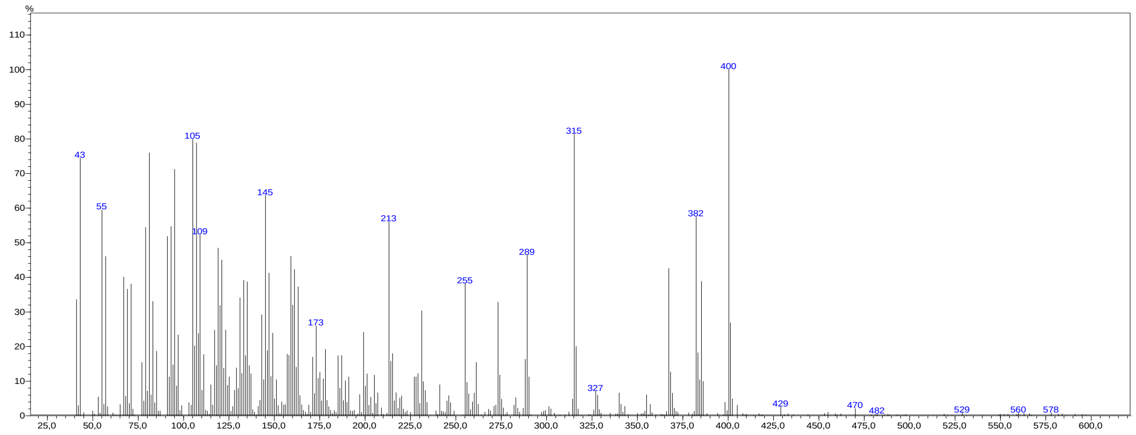

Figure 5: RMN ^{1}H NMR spectrum (DMSO-d_6, 500MHz) of compounds **4.**

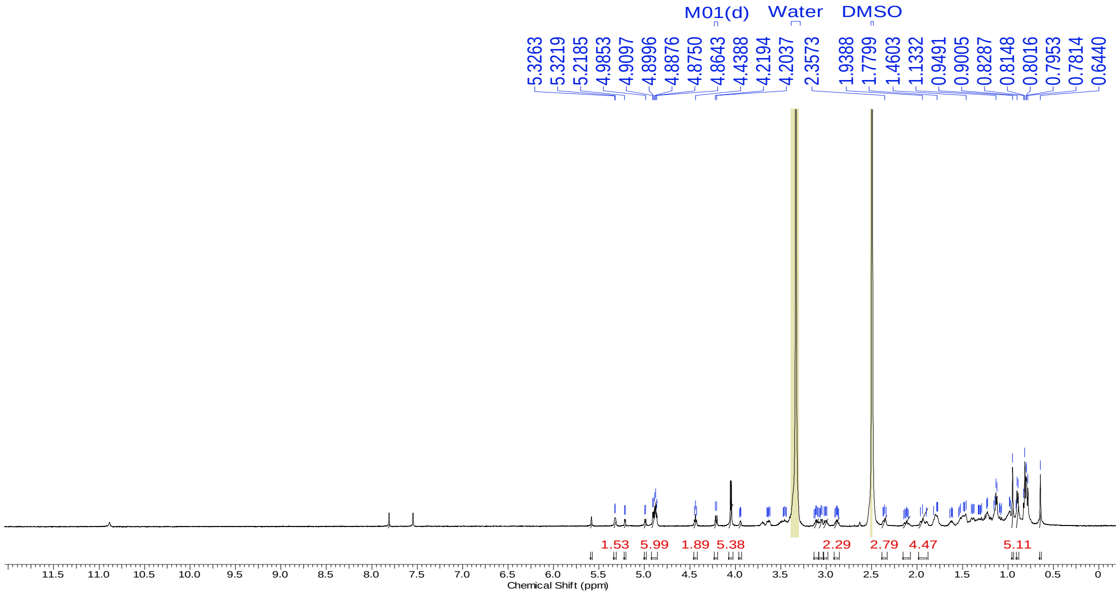

Figure 6. Expansion of the ^{1}H NMR spectrum (DMSO-d_6, 500MHz) of compounds **4** (δH 3.40 – 5.50 ppm).

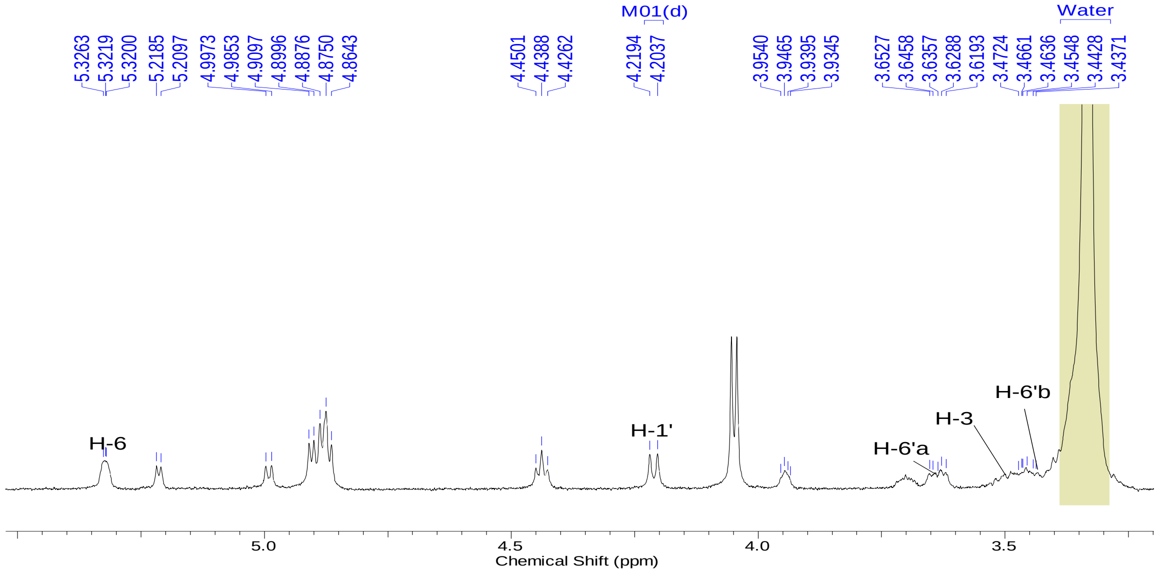

Figure 7. Expansion of the ^{1}H NMR spectrum (DMSO-d_6, 500MHz) of compounds **4** (δH 0.5 – 3.50 ppm).

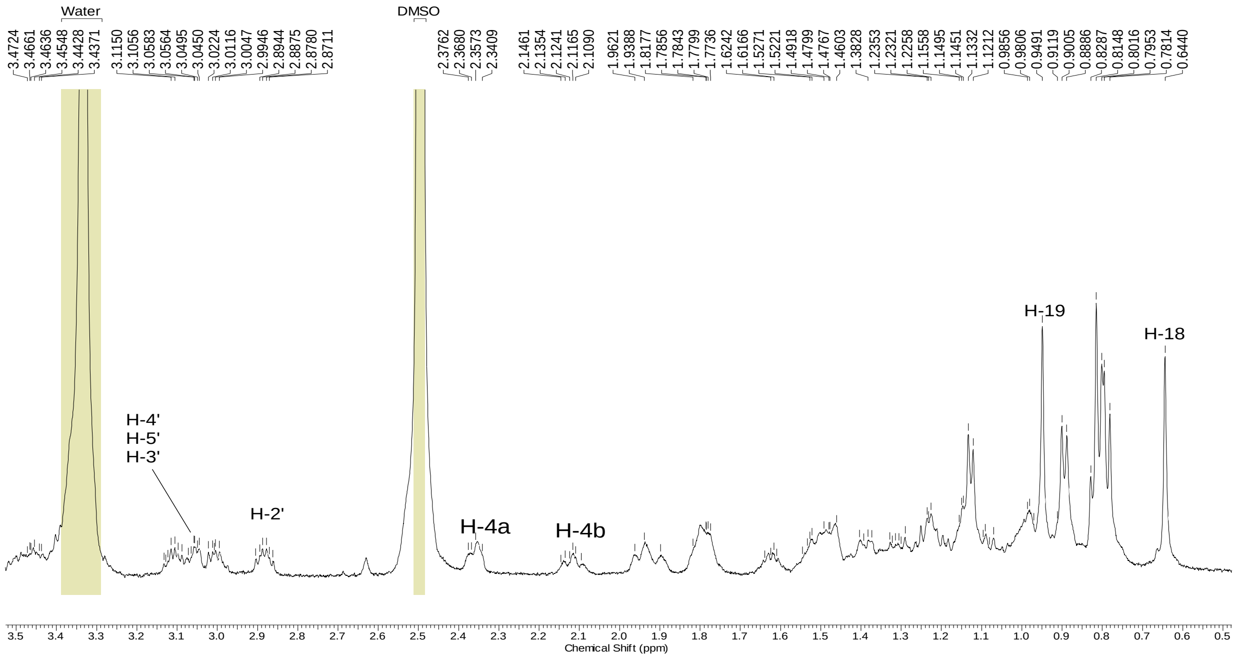

Figure 8. DEPTQ NMR spectrum (DMSO-d_6, 125 MHz) of compounds **4.**

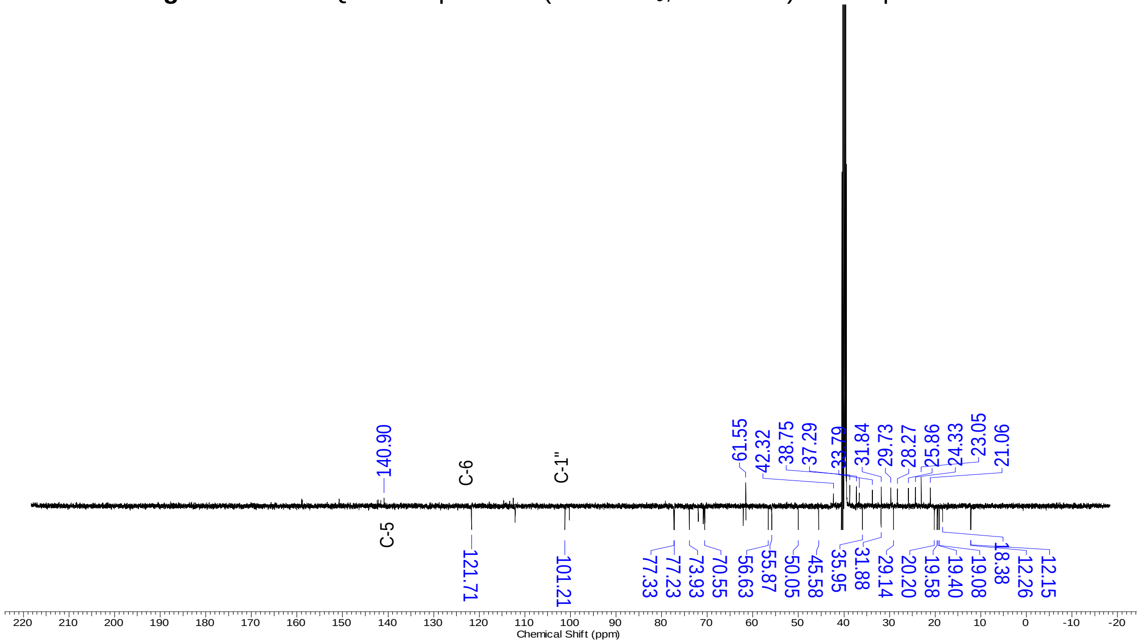

Figure 9. RMN ^{1}H NMR spectrum ($CDCl_3$, 500MHz) and expansions of compounds **5.**

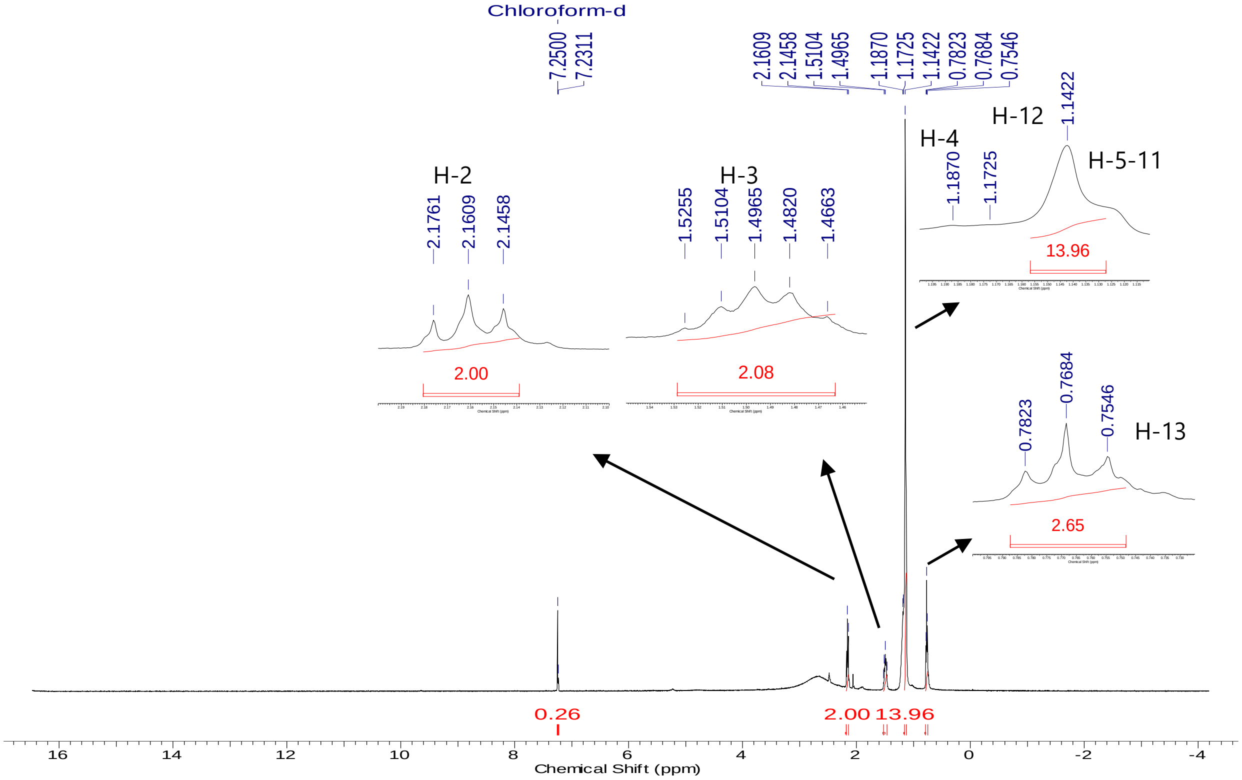

Figure 10. DEPTQ NMR spectrum ($CDCl_3$, 125 MHz) and expansion of compound **5.**

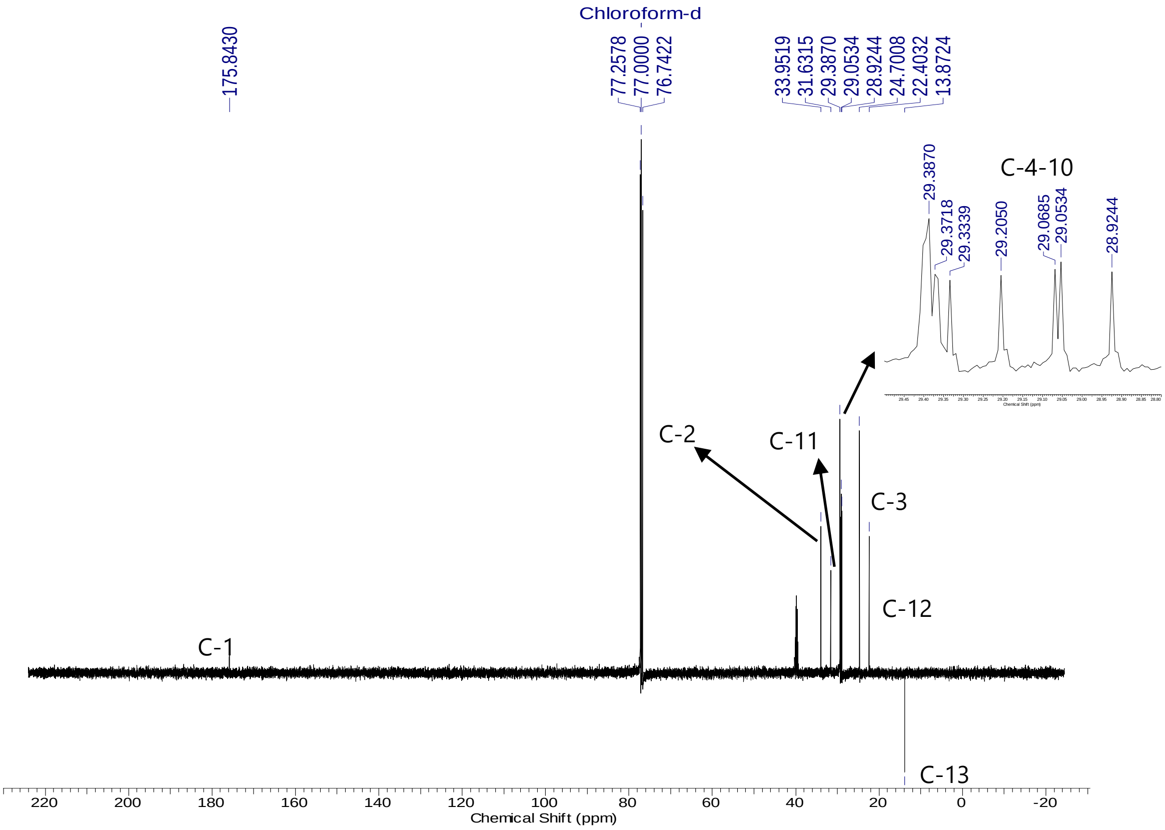

Figure11. ^{1}H NMR spectrum and its expansions ($CDCl_3$, 500 MHz) of compound **6.**

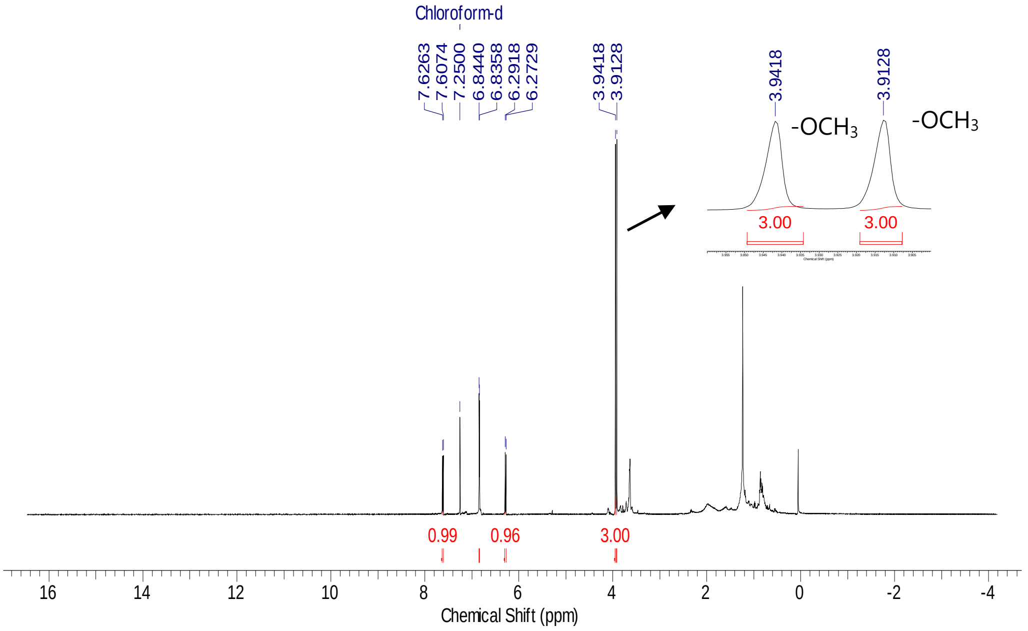

Figure 12. Expansion of the ^{1}H NMR spectrum ($CDCl_3$, 500MHz) of compound **6** (δH 6.60 – 7.70 ppm).

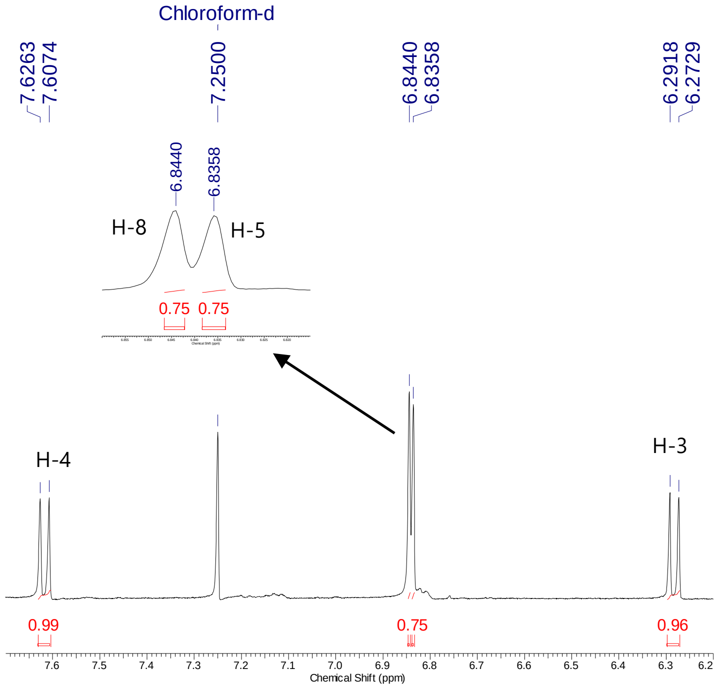

Figure 13. DEPTQ NMR spectrum ($CDCl_3$, 125 MHz) of compound **6.**

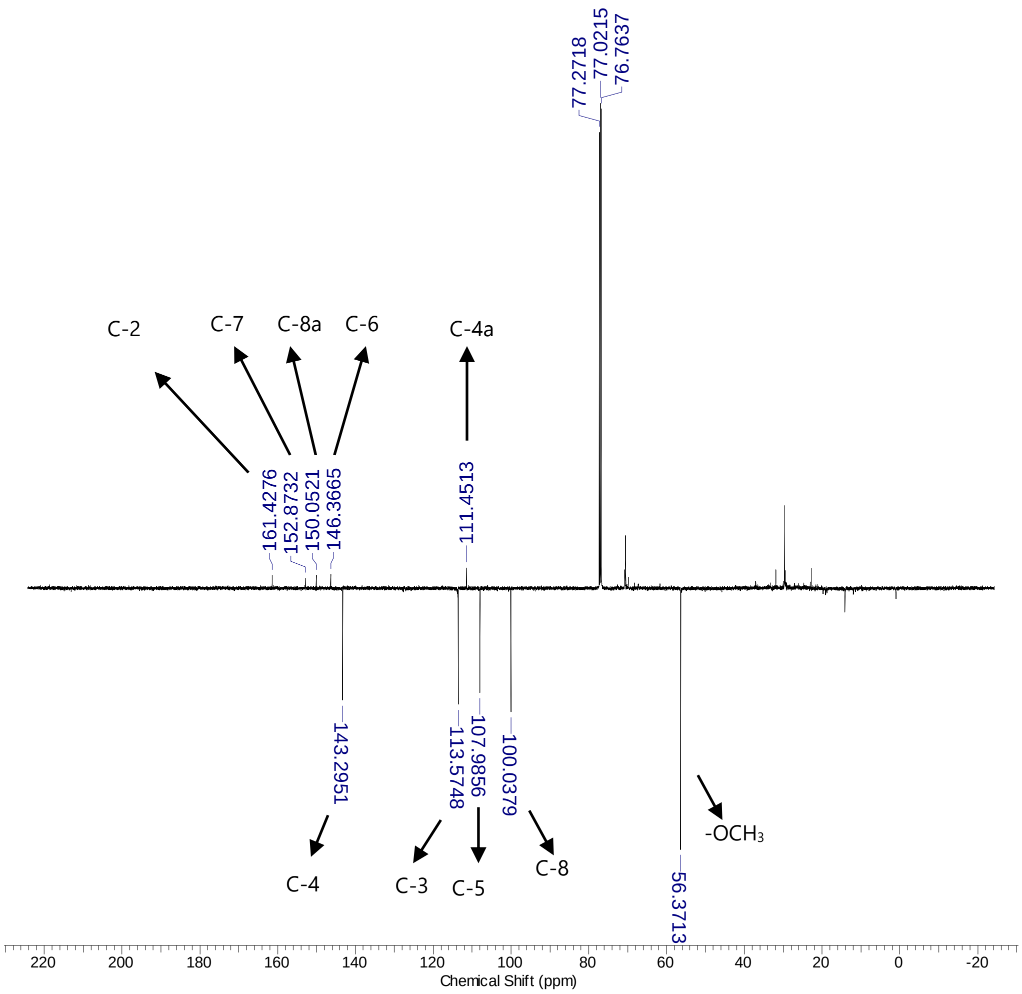

Figure14. ^1H spectrum (CD_3OD, 500 MHz) of compound **7.**

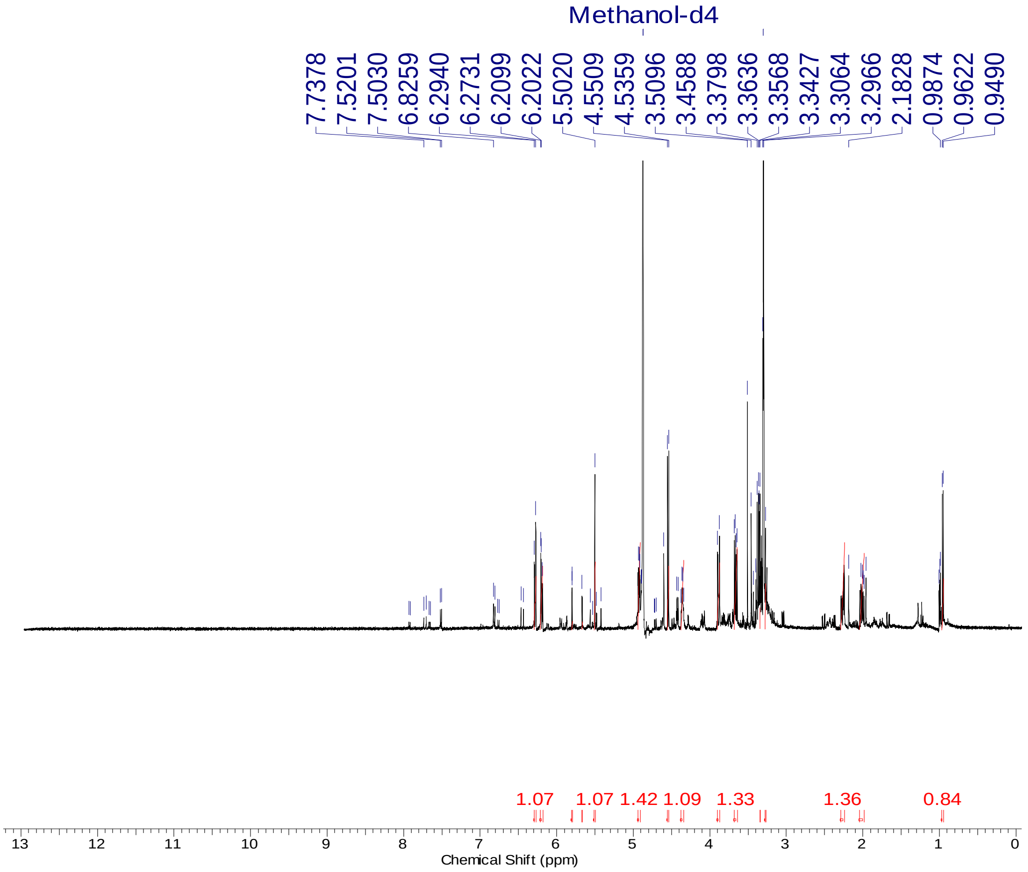

Figure 15. Expansion of the ^1H spectrum (CD_3OD, 500 MHz) of compound **7** (δH 1.92 – 2.30 ppm).

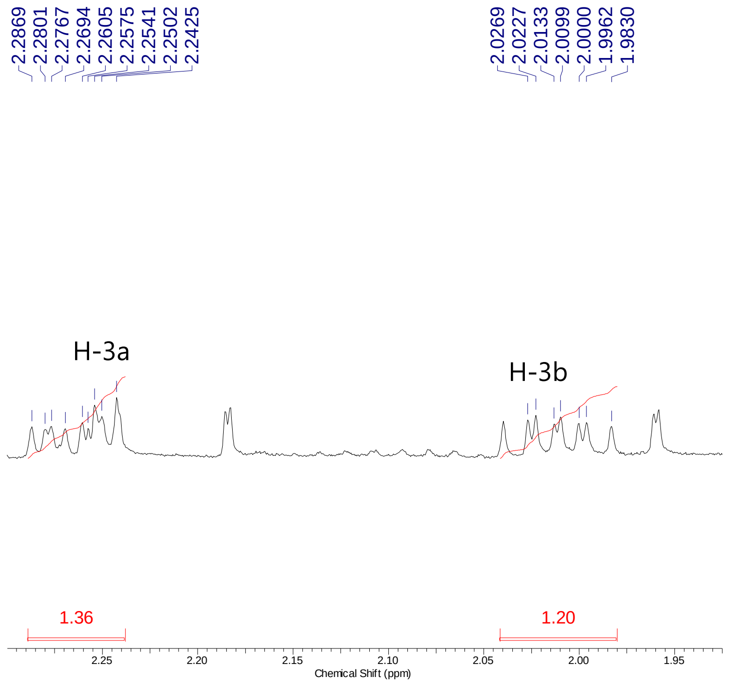

Figure 16. Expansion of the ^{1}H spectrum (CD_3OD, 500 MHz) of compound **7** (δH 3.62 – 3.92 ppm).

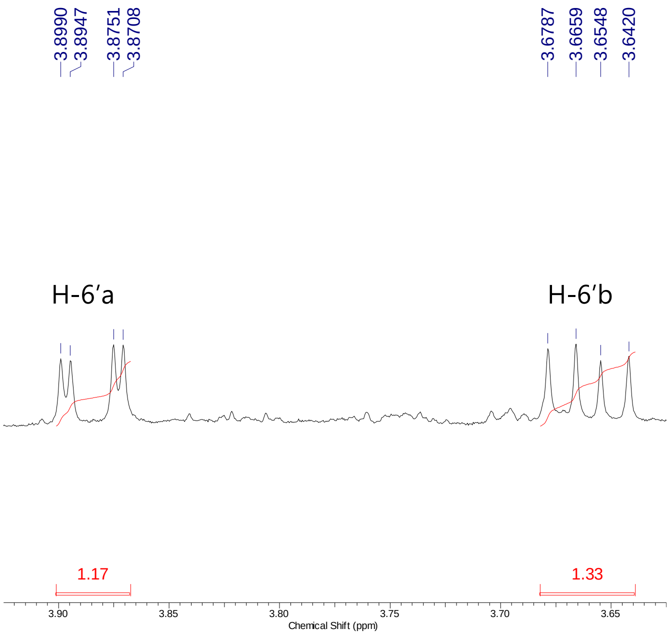

Figure 17. Expansion of the ^{1}H spectrum (CD_3OD, 500 MHz) of compound **7** (δH 3.25 – 3.40 ppm).

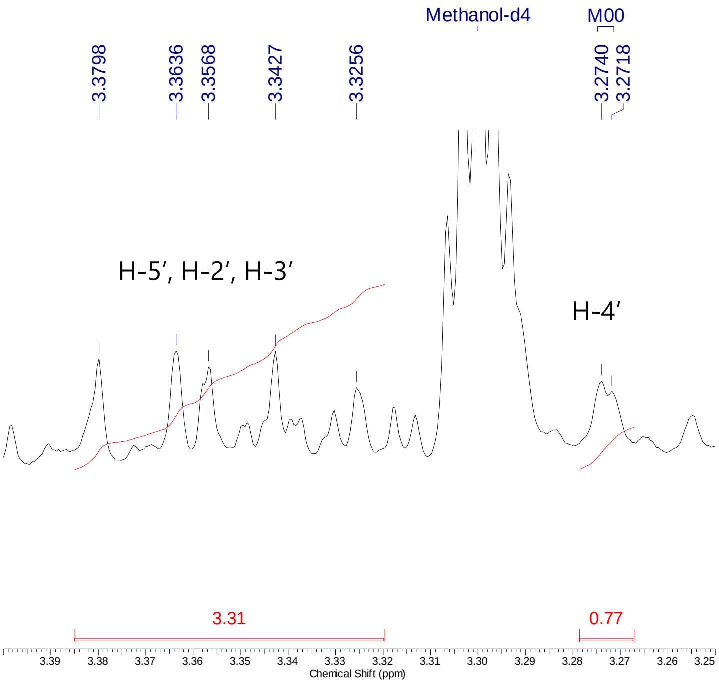

Figure 18. Expansion of the ¹H spectrum (CD_3OD, 500 MHz) of compound **7** (δH 4.30 – 4.67 ppm).

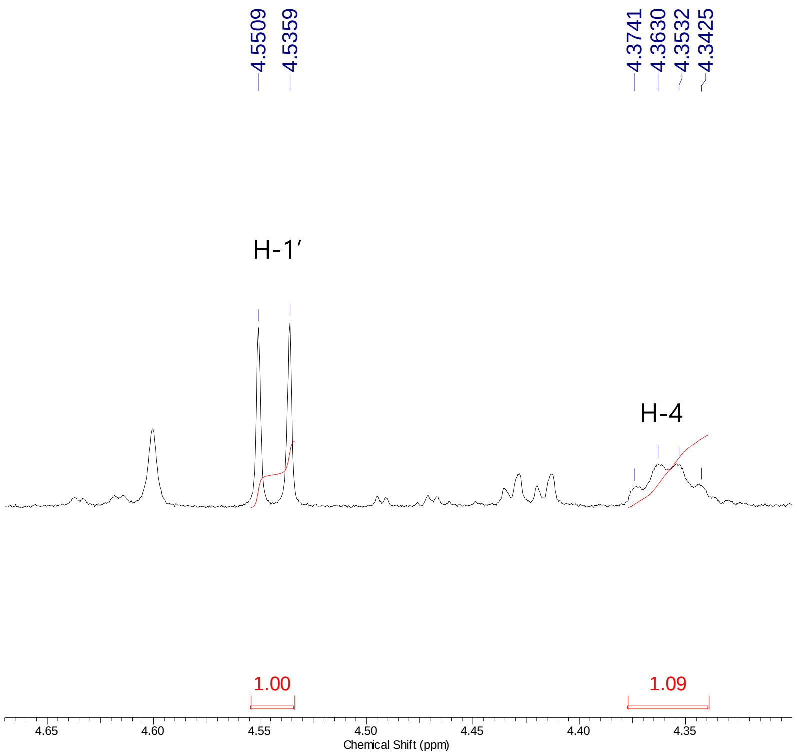

Figure 19. Expansion of the ¹H spectrum (CD_3OD, 500 MHz) of compound **7** (δH 4.90 – 5.55 ppm).

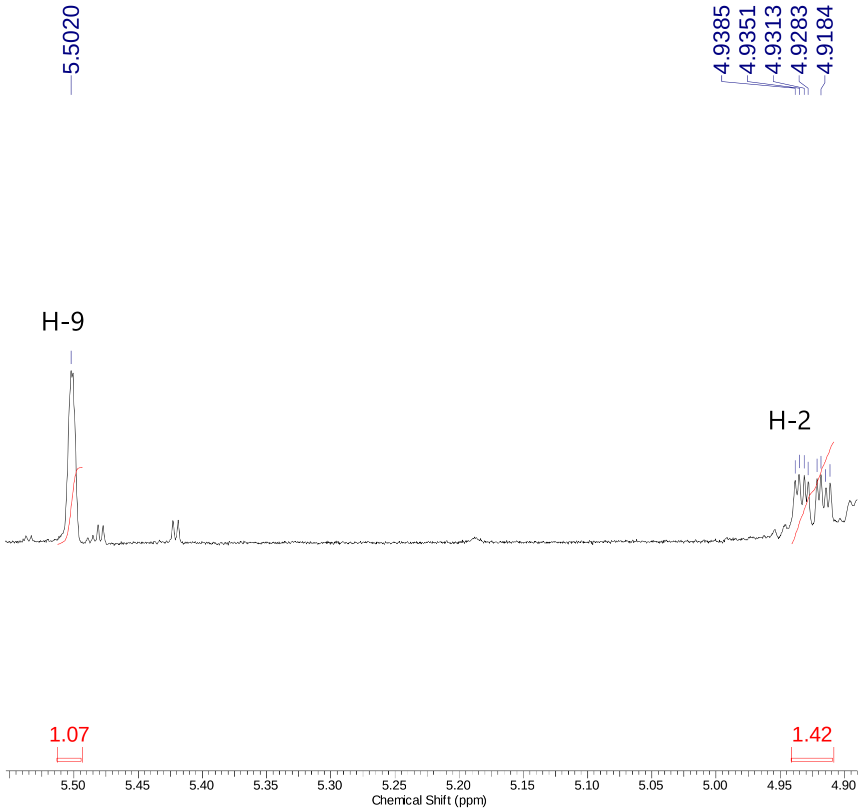

Figure 20. Expansion of the ^{1}H spectrum (CD_3OD, 500 MHz) of compound **7** (δH 6.15 – 6.32 ppm).

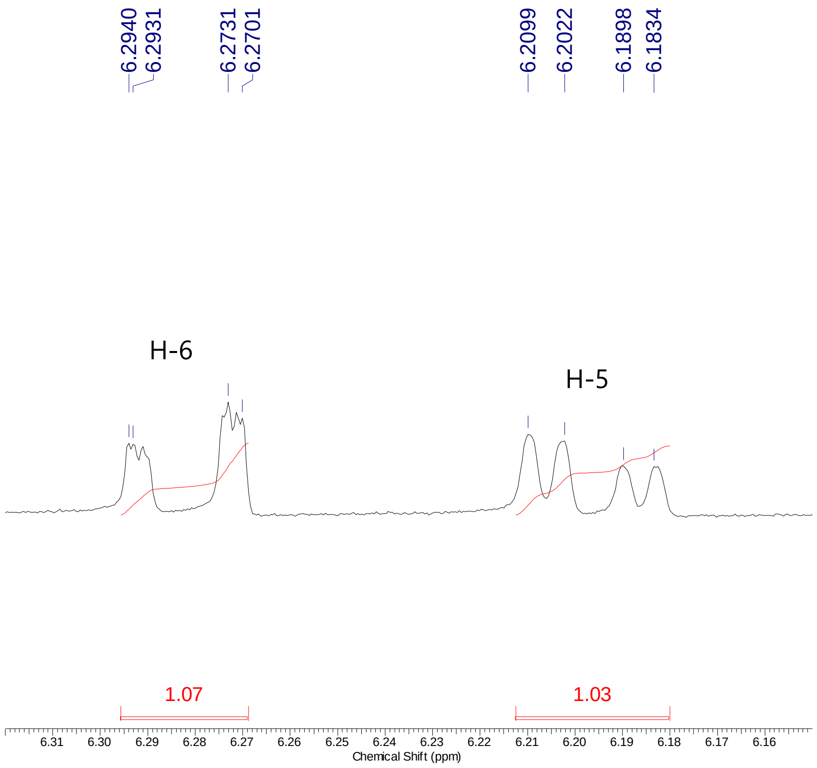

Figure 21. ^{13}C spectrum (CD_3OD, 125 MHz) of compound **7.**

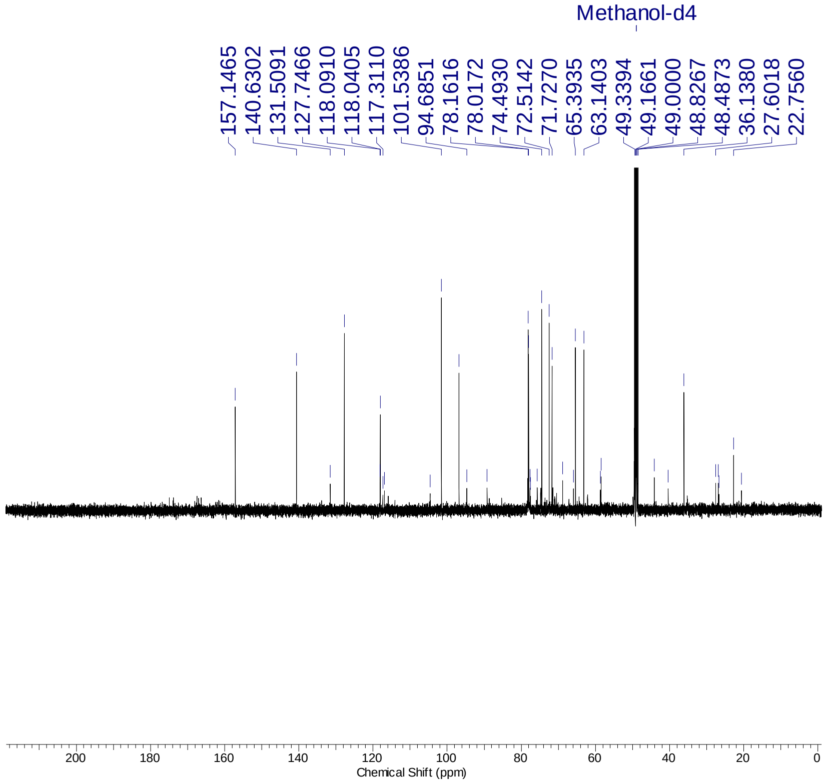

Figure 22. Expansion of the ^{13}C spectrum (CD_3OD, 125 MHz) of compound **7** (δC 35.0 - δC 102.0 ppm).

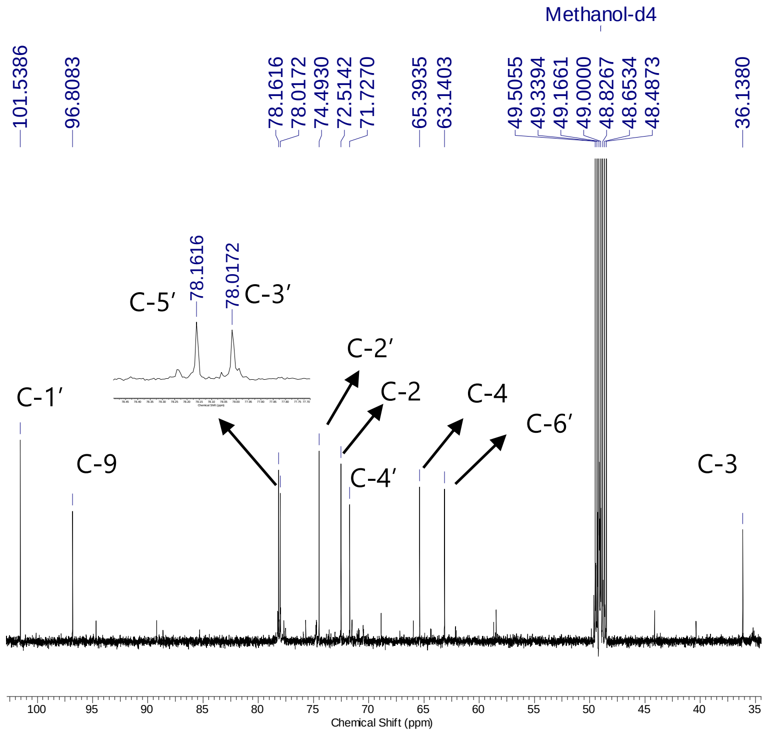

Figure 23. Expansion of the ^{13}C spectrum (CD_3OD, 125 MHz) of compound **7** (δC 117.0 - δC 158.0 ppm).

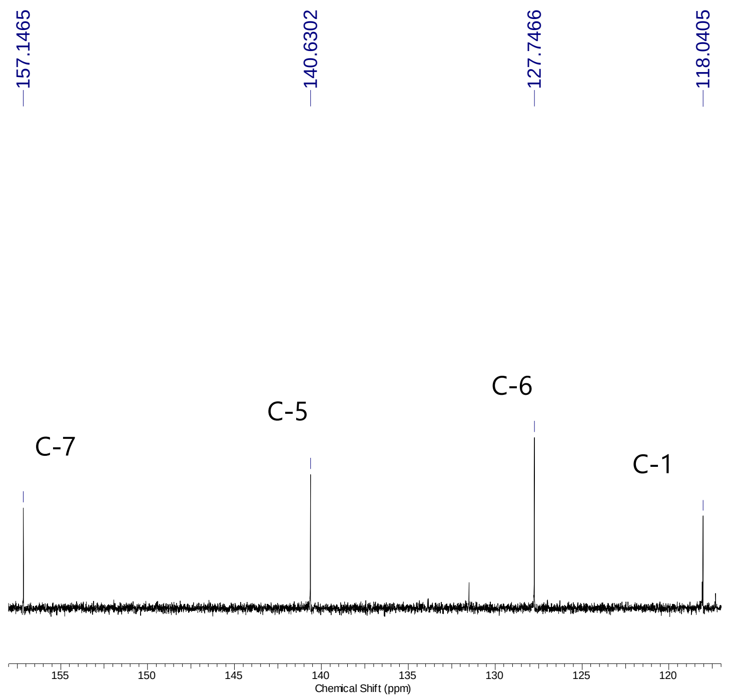

Figure 24. COSY spectrum (CD_3OD, 500 MHz) of compound **7.**

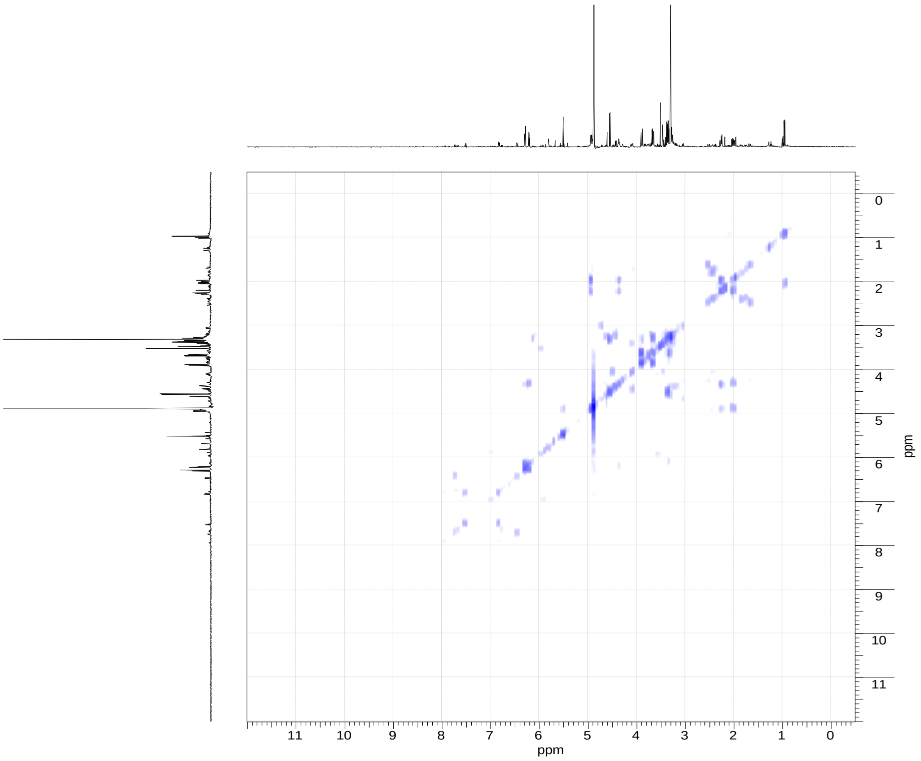

Figure 25. HSQC spectrum (CD_3OD, 125/ 500 MHz) of compound **7.**

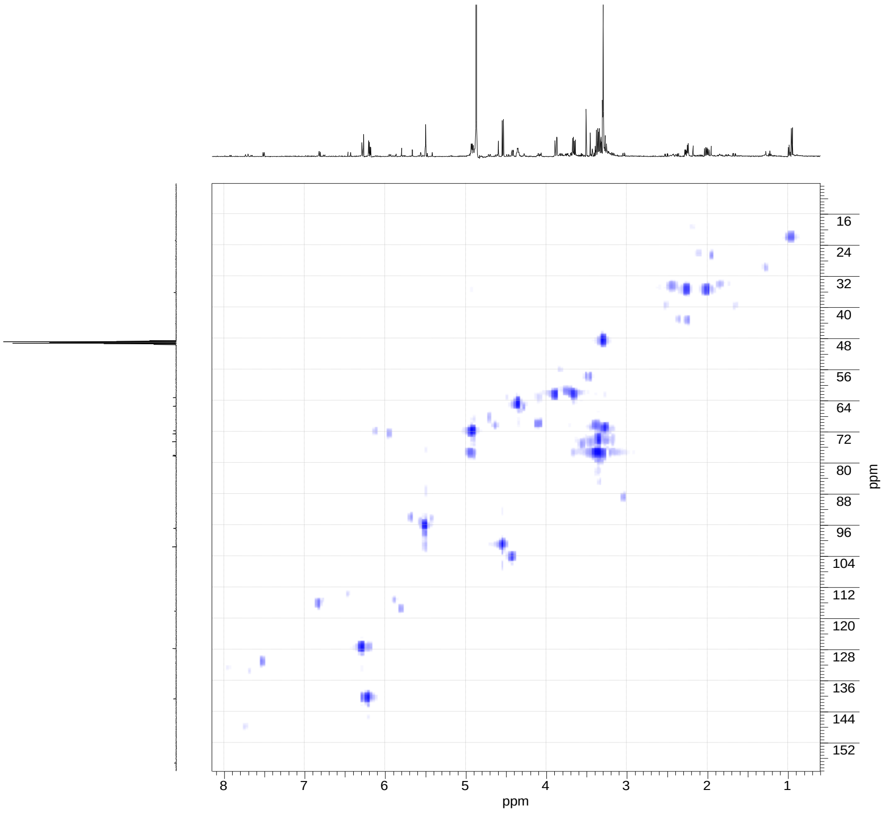

Figure 26. HMBC spectrum (CD_3OD, 125/ 500 MHz) of compound **7.**

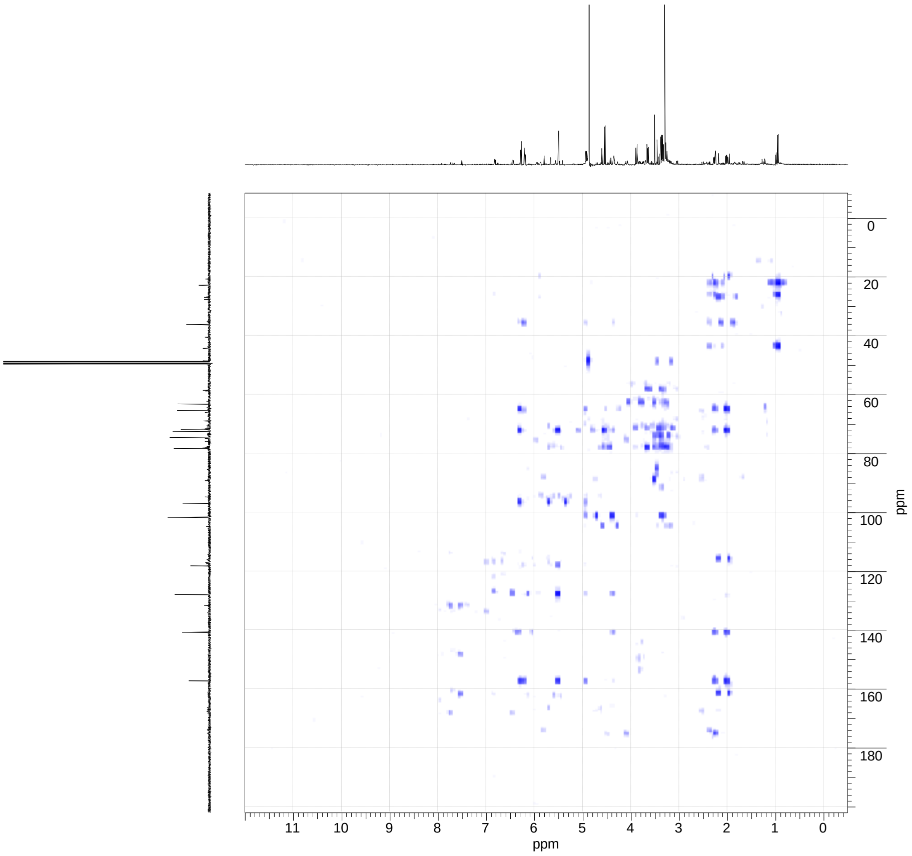

Supplement: Supplementary file 1 — Supporting file 1: cbdv70790‐sup‐0001‐SuppMat.pdf [file CBDV-23-e02503-s001.pdf]
